# Supplementary material for: Complexome profiling of the Chlamydomonas psb28 mutant reveals TEF5 as an early PSII assembly factor
Source: Plant Cell. 2025 Mar 17;37(6):koaf055. doi: 10.1093/plcell/koaf055 (PMC12164590; doi:10.1093/plcell/koaf055)
Supplement: koaf055_Supplementary_Data [file koaf055_supplementary_data.zip › psb28_interactive.zip!/plots/intensity/qualityPlots/peptides/ATO1_Cre17.g723650.t1.2.html]

### Cre17.g723650.t1.2

Trivial name: ATO1  
SeqCoverage [median]: 41.00  
Mass: 47.36 kDa  
  
 

| Sequence | MissedCleavage | Mass [Da] | wtIntensity | muIntensity | Score |
| --- | --- | --- | --- | --- | --- |
| AFAAVGVDPAIMGVGPAVAIPAAVAR | 0 | 2390.31 | 7430.28 | 0.00 | 111.83 |
| AVAGPQQLLSK | 0 | 1110.64 | 5508.89 | 10116.57 | 111.61 |
| AVAGPQQLLSKDAVV | 1 | 1494.84 | 947.77 | 1311.55 | 58.13 |
| DTDAADLLSTLFK | 0 | 1408.71 | 3200.37 | 0.00 | 163.34 |
| FKDEIVPVATK | 1 | 1245.70 | 1251.53 | 5411.55 | 84.66 |
| GGLRDTDAADLLSTLFK | 1 | 1791.94 | 492.53 | 0.00 | 56.96 |
| GLPILGVFR | 0 | 970.60 | 4213.67 | 1991.86 | 89.90 |
| GSTTMETLGALK | 0 | 1207.61 | 822.15 | 0.00 | 90.15 |
| IASFFAGIPDVVPVR | 0 | 1586.88 | 5602.56 | 0.00 | 174.10 |
| ITISEDDGIR | 0 | 1117.56 | 0.00 | 3665.83 | 79.47 |
| ITISEDDGIRGSTTMETLGALK | 1 | 2307.16 | 0.00 | 5891.31 | 58.49 |
| KTQDEFAVR | 1 | 1092.56 | 516.93 | 879.63 | 90.91 |
| LGLDEAK | 0 | 744.40 | 1987.40 | 0.00 | 79.45 |
| NTADDDVVIVASLR | 0 | 1486.76 | 3462.64 | 3993.01 | 111.39 |
| RNTADDDVVIVASLR | 1 | 1642.86 | 1867.11 | 7363.81 | 90.37 |
| TGVEPQAIGDIVIGSVLGPSSQR | 0 | 2279.21 | 6560.05 | 0.00 | 131.59 |
| TQDEFAVR | 0 | 964.46 | 7773.66 | 9478.87 | 169.09 |
| VNPNGGAIALGHPLGATGAR | 0 | 1841.99 | 464.62 | 2395.89 | 76.06 |
|  |  |  | 52102.1 | 52499.9 |  |
